# Complete plastome sequence of *Xylosma longifolia* Clos. (Salicaceae)

**DOI:** 10.1080/23802359.2021.1899870

**Published:** 2021-03-18

**Authors:** Peng Chen, Xiao-Feng Zhang, Jacob B. Landis, Zhi-Xin Zhu, Hua-Feng Wang

**Affiliations:** a Hainan Key Laboratory for Sustainable Utilization of Tropical Bioresources, College of Tropical Crops, Hainan University, Haikou, China; b School of Integrative Plant Science, Section of Plant Biology and the L.H. Bailey Hortorium, Cornell University, Ithaca, NY, USA

**Keywords:** *Xylosma longifolia*, Salicaceae, plastome, genome structure, phylogenetic

## Abstract

*Xylosma longifolia* is a tree species within Salicaceae and is distributed in Guizhou, Yunnan, Fujian, Guangxi, Guangdong, and Hainan provinces of China as well as in Vietnam, Laos, and India. There are no studies utilizing the complete plastome of *Xylosma longifolia* in the current literature. Therefore, this report provides a reference for the plastid gene sequence of *Xylosma longifolia*, and it contributes to the phylogenetic placement and species identification. In this report, we described the complete plastome sequence of *Xylosma longifolia.* The complete plastome length of *Xylosma longifolia* is 156,938 bp and has the typical quadripartite structure and gene content of angiosperms, including two inverted repeat (IR) regions of 27,514 bp, a large single-copy (LSC) region of 85,221 bp and a small single-copy (SSC) region of 16,689 bp. The plastome contains 130 genes, including 86 protein coding genes, 36 tRNA genes, eight rRNA genes (5S rRNA, 4.5S rRNA, 16S rRNA, and 23S rRNA). The GC content of the plastome is 36.8%. The complete plastome sequence will be a valuable resource for studies involving the phylogenetic inference of Salicaceae.

*Xylosma longifolia* is a tree species within Salicaceae, occurring in Guizhou, Yunnan, Fujian, Guangxi, Guangdong, and Hainan provinces of China as well as in Vietnam, Laos, and India. *Xylosma longifolia* is a small evergreen tree or shrub, growing to approximately four to seven meters in height with grayish brown bark. Its leaves and barks could be used as medicine. Compared to the nuclear genome, chloroplast genomes (plastomes) have several advantages including a haploid nature, maternal inheritance, conserved typical structure in vascular plants, and minimal gene duplications (Wang et al. 2020). Complete plastome sequences have become a powerful tool for resolving plant phylogenies. Therefore, the study of plastomes has significant benefits for species identification and systematic position. However, there are no reports of the complete plastome sequence of *X. longifolia.*

In this report, we describe the complete plastome sequence of *X. longifolia* (GenBank accession number: MW357610) for promoting the protection of germplasm and providing useful genomic resources. The plant sample of *X. longifolia* was collected from Forest Park, Wanding Town, Ruili county, Yunnan Province, China (100.22°E, 26.87°N). The voucher specimen (voucher code, RL0607) and associated DNA were deposited in the Herbarium of the Institute of Herbarium of China National GeneBank (code of herbarium: HCNGB).

We assembled approximately six Gb of clean data using the plastome of *Populus alba* (GenBank accession number: AP008956) (Rivarola et al. 2011) as a reference with MITO bim v1.8 (Hahn et al. 2013). We aligned all assembled contigs to the reference with BLAST as implemented in Geneious R11.0.5 (Biomatters Ltd., Auckland, New Zealand) followed by mapping clean reads to the assembly to verify sequencing depth and contig overlap using Geneious R11.0.5. The assembled plastome of *Xylosma longifolia* was annotated with DOGMA (Dual Organellar Genome Annotator). Intron/exon boundaries were further determined with alignments in MAFFT v7 against the reference plastomes of *Populus alba* (AP008956). The annotated plastomes sequences were deposited in GenBank with accession number (MW357610). We used OGDRAW to draw genome maps with subsequent manual editing. Sequences of *Xylosma longifolia* and related species were aligned using PROGRESSIVEMAUVE v2.4.0 to compare the structure and gene content among the plastomes.

The results showed that the length of the plastome is 156,938 bp, which has the typical quadripartite structure of angiosperms, including two inverted repeats (IR), one large single copy (LSC) region and one small single copy (SSC) region. The plastome contains 130 genes, including 79 unique protein coding genes (seven of which are repeated in the IR), 29 unique tRNA genes (seven of which are repeated in the IR), and eight rRNA genes (5S rRNA, 4.5 s rRNA, 16S rRNA and 23S rRNA are repeated in the IR). The GC content of the plastome overall is 36.8%, with the LSC, SSC, and IRs having a GC content of 34.5%,42.1%, and 42.1%, respectively.

We used RaxML with 1000 bootstraps and the GTRGAMMAI substitution model, infer the Maximum likelihood (ML) phylogenetic tree of twelve published complete plastomes of Malpighiales, using *Euphorbia tirucalli* NC_042193.1, *Euphorbia smithii* MN646684.1, and *Euphorbia esula* NC_033910.1 as outgroups. By reconstructing the phylogenetic relationships of *X. longifolia* and published plastomes of related taxa in the order, we found that *X. longifolia* is most closely related to *Flacourtia indica* and *F. jangomas* within Salicaceae (Figure 1). Most nodes in the inferred ML trees were highly supported, providing an initial beneficial understanding to the phylogenetic relationships of Salicaceae. After comparing the results obtained here with a previously published phylogeny of *X. longifolia* (e.g. Rivarola et al. 2011), we find that the phylogenetic resolution is improved over previous inferred trees relying on few plastid or nuclear markers (e.g. Hamzeh and Dayanandan 2004; Liu et al. 2016). Our results demonstrate the power of plastomes in phylogenomics to improve estimates of phylogeny among genera and subfamilies, and it provides new insights into plastome evolution within Salicaceae. The complete plastome sequence of *Xylosma longifolia* will provide valuable genetic information for the protection of this species along with the phylogenetic study for Salicaceae.

**Figure 1.**
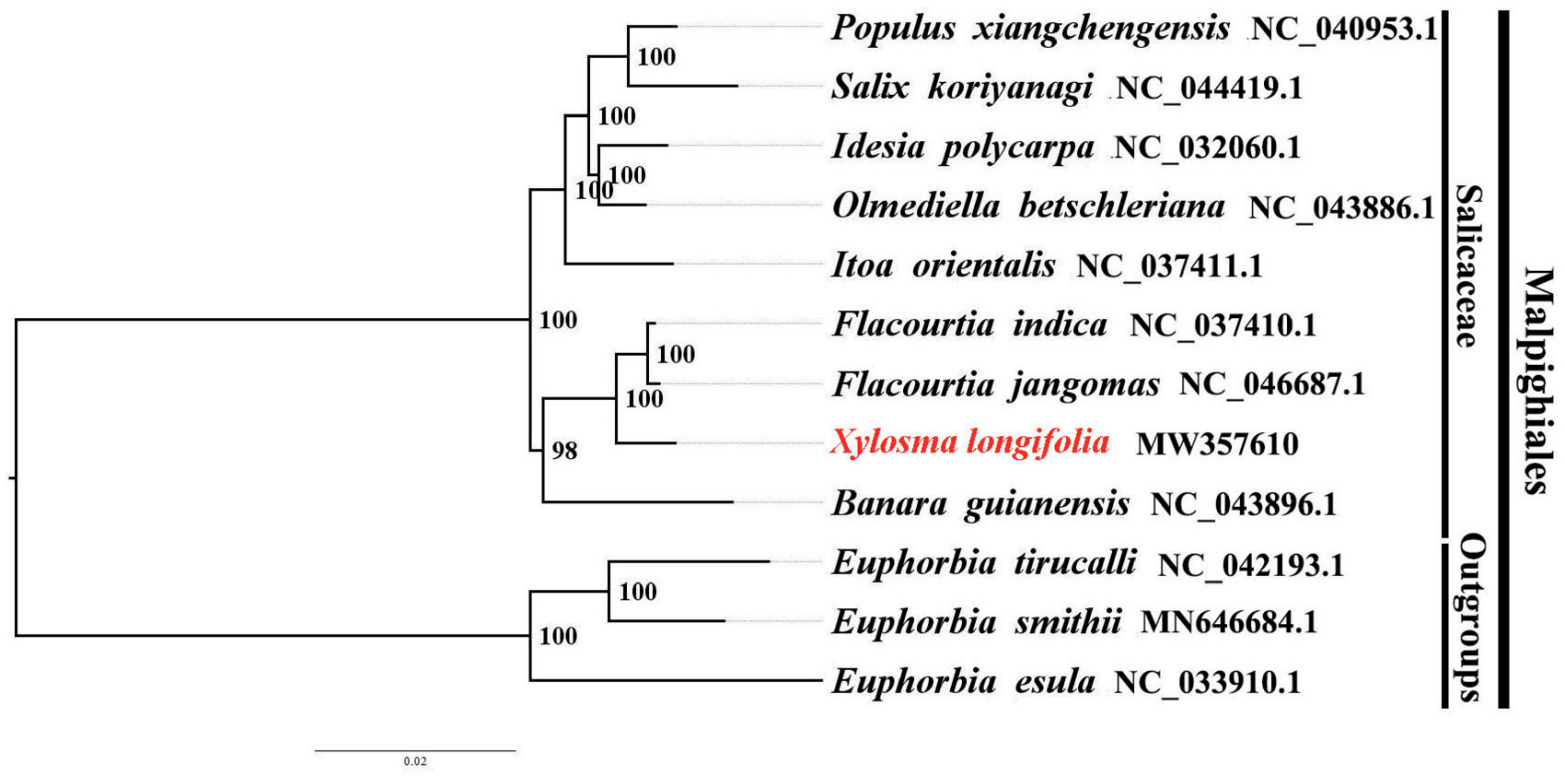
The ML phylogeny inferred from12 complete plastome sequences using RAxML. *Xylosma longifolia* (GenBank accession number, MW357610, this study), *Populus xiangchengensis* NC040953.1; *Salix koriyanagi.*NC044419.1; *Idesia polycarpa* NC032060.1; *Olmediella betschleriana* NC043886.1; *Itoa orientalis* NC037411.1; *Flacourtia indica* NC037410.1; *Flacourtia jangomas* NC046687.1; *Banara guianensis* NC_043896.1; Outgroups: *Euphorbia tirucalli* NC042193.1; *Euphorbia smithii* MN646684.1; *Euphorbia esula* NC033910.1.

## Data Availability

The genome sequence data supporting the results of this study are publicly available on GenBank of NCBI (https://www.ncbi.nlm.nih.gov/) with registration number MW357610. The associated SRA, BioProject and Bio-Sample numbers are SRS3260867, PRJNA438407 and SAMN08770789, respectively.
